# Crystal structure of 1-isopropyl-4,7-dimethyl-3-nitro­naphthalene

**DOI:** 10.1107/S2056989015014395

**Published:** 2015-08-15

**Authors:** Ahmed Benharref, Jamal Elkarroumi, Lahcen El Ammari, Mohamed Saadi, Moha Berraho

**Affiliations:** a Laboratoire de Chimie des Substances Naturelles, "Unité Associé au CNRST (URAC16)", Faculté des Sciences Semlalia, BP 2390 Bd My Abdellah, 40000 Marrakech, Morocco; b Laboratoire de Chimie du Solide, Appliquée, Faculté des Sciences, Université Mohammed V, Avenue Ibn Battouta, BP 1014, Rabat, Morocco

**Keywords:** crystal structure, essential oil of the Atlas cedar, nitro-naphthalene, C—H⋯O inter­action

## Abstract

The title compound, C_15_H_17_NO_2_, was synthesized from a mixture of α-himachalene (2-methyl­ene-6,6,9-tri­methylbi­cyclo­[5.4.0^1,7^]undec-8-ene) and β-himachalene (2,6,6,9-tetra­methylbi­cyclo­[5.4.0^1,7^]undeca-1,8-diene), which were isolated from an oil of the Atlas cedar (*Cedrus Atlantica*). The naphthalene ring system makes dihedral angles of 68.6 (2) and 44.3 (2)°, respectively, with its attached isopropyl C/C/C plane and the nitro group. In the crystal, mol­ecules held together by a C—H⋯O inter­action, forming a chain along [-101].

## Related literature   

For the main constituents of the essential oil of the Atlas cedar, see: El Haib *et al.* (2011 ▸); Loubidi *et al.* (2014 ▸). For the reactivity of these sesquiterpenes and their derivatives, see: Oukhrib *et al.* (2013 ▸); Zaki *et al.* (2014 ▸); Benharref *et al.* (2015 ▸). For anti­fungal activity of these sesquiterpenes and derivatives, see: Daoubi *et al.* (2004 ▸).
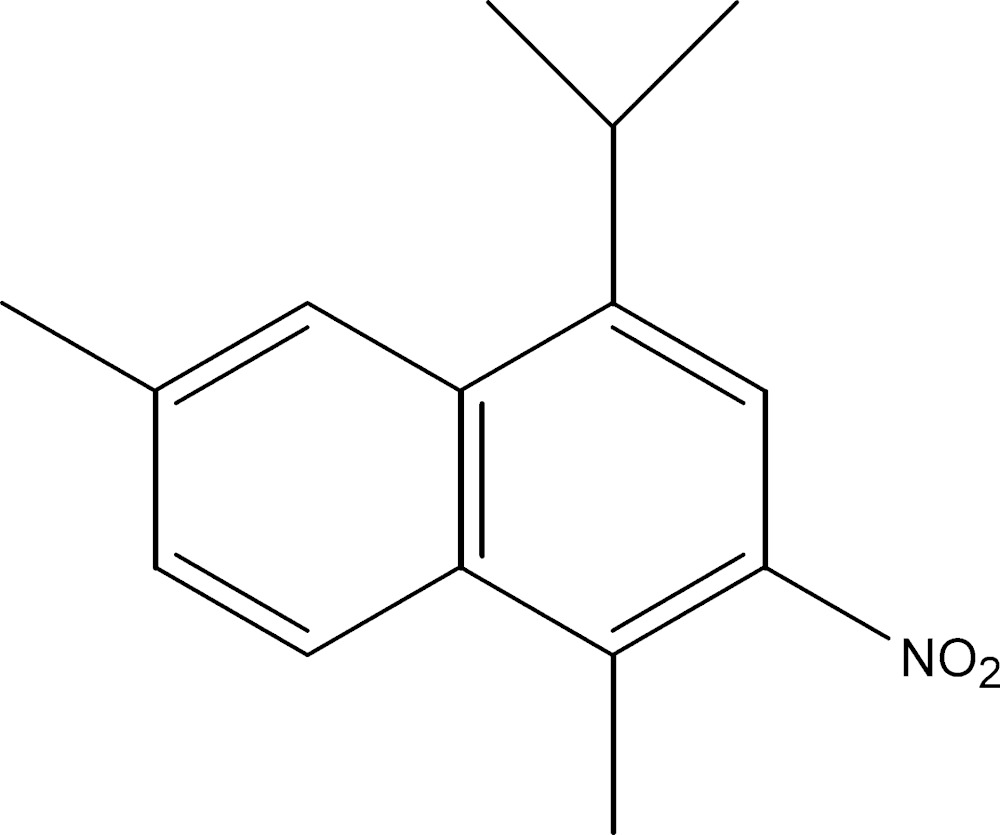


## Experimental   

### Crystal data  

C_15_H_17_NO_2_
*M* *_r_* = 243.30
Monoclinic, 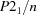

*a* = 9.7637 (7) Å
*b* = 12.6508 (9) Å
*c* = 11.6162 (8) Å
β = 113.897 (2)°
*V* = 1311.82 (16) Å^3^
*Z* = 4
Mo *K*α radiation
μ = 0.08 mm^−1^
*T* = 296 K
0.45 × 0.35 × 0.30 mm

### Data collection  

Bruker APEXII CCD diffractometer
Absorption correction: multi-scan (*SADABS*; Bruker, 2009 ▸) *T*_min_ = 0.652, *T*_max_ = 0.746
21437 measured reflections
2686 independent reflections
2164 reflections with *I* > 2σ(*I*)
*R*_int_ = 0.027

### Refinement  

*R*[*F* ^2^ > 2σ(*F* ^2^)] = 0.047
*wR*(*F* ^2^) = 0.147
*S* = 1.07
2686 reflections
167 parameters
H-atom parameters constrained
Δρ_max_ = 0.22 e Å^−3^
Δρ_min_ = −0.17 e Å^−3^

### 

Data collection: *APEX2* (Bruker, 2009 ▸); cell refinement: *SAINT* (Bruker, 2009 ▸); data reduction: *SAINT*; program(s) used to solve structure: *SHELXS97* (Sheldrick, 2008 ▸); program(s) used to refine structure: *SHELXL2013* (Sheldrick, 2015 ▸); molecular graphics: *ORTEP-3 for Windows* (Farrugia, 2012 ▸); software used to prepare material for publication: *WinGX* (Farrugia, 2012 ▸).

## Supplementary Material

Crystal structure: contains datablock(s) I. DOI: 10.1107/S2056989015014395/is5409sup1.cif

Structure factors: contains datablock(s) I. DOI: 10.1107/S2056989015014395/is5409Isup2.hkl

Supporting information file. DOI: 10.1107/S2056989015014395/is5409Isup3.cml

**Fig. 1.**
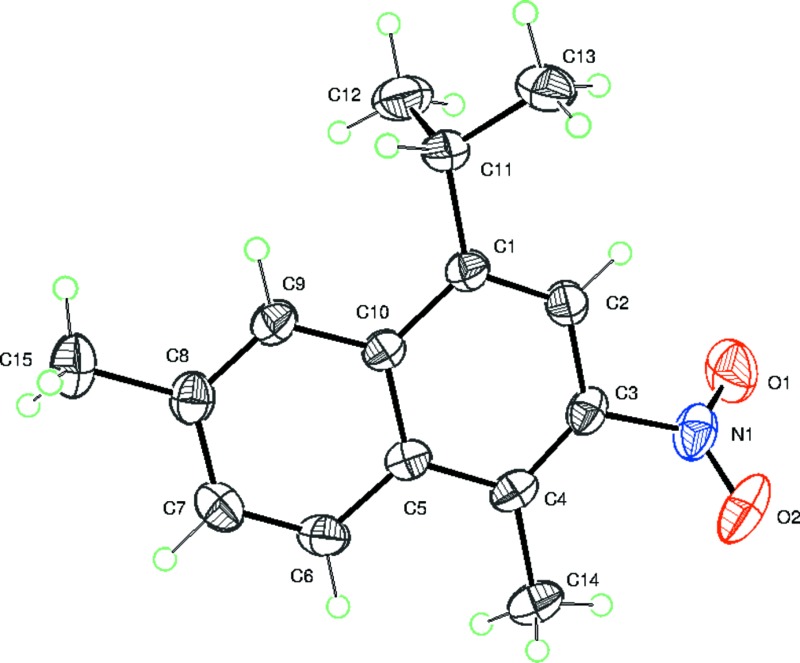
: Molecular structure of the title compound with the atom-labelling scheme. Displacement ellipsoids are drawn at the 30% probability level. H atoms are represented as small spheres of arbitrary radii.

**Fig. 2.**
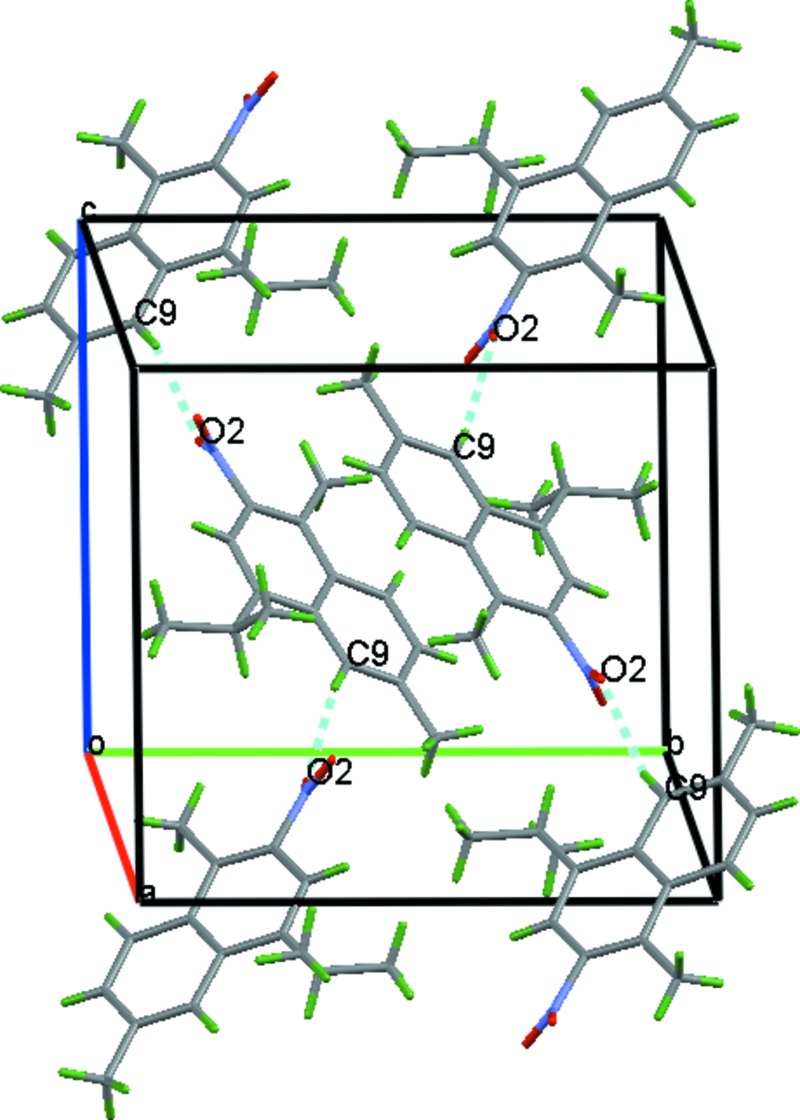
Partial packing view showing the C—H···O interactions (dashed lines) and the formation of a chain along the ac diagonal.

. DOI: 10.1107/S2056989015014395/is5409fig1.tif
Mol­ecular structure of the title compound with the atom-labelling scheme. Displacement ellipsoids are drawn at the 30% probability level. H atoms are represented as small spheres of arbitrary radii.

ac . DOI: 10.1107/S2056989015014395/is5409fig2.tif
Partial packing view showing the C—H⋯O inter­actions (dashed lines) and the formation of a chain along the *ac* diagonal.

CCDC reference: 1415866

Additional supporting information:  crystallographic information; 3D view; checkCIF report

## Figures and Tables

**Table 1:** Hydrogen-bond geometry (, )

*D*H*A*	*D*H	H*A*	*D* *A*	*D*H*A*
C9H9O2^i^	0.93	2.60	3.4823(18)	159

Symmetry code: (i) 
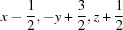
.

## supplementary crystallographic information

### S1. Comment

The bicyclic sesquiterpenes, *α*- and *β*-himachalene, are the main constituents of the essential oil of the Atlas cedar (*Cedrus Atlantica*) (El Haib *et al.*, 2011; Loubidi *et al.*, 2014). The reactivity of these sesquiterpenes and its derivatives has been studied extensively by our team in order to prepare new products having biological proprieties (Oukhrib *et al.*, 2013; Zaki *et al.*, 2014; Benharref *et al.*, 2015). Indeed, these compounds were tested, using the food poisoning technique, for their potential antifungal activity against the phytopathogen Botrytis cinerea (Daoubi *et al.*, 2004).

The catalytic dehydrogenation of the mixture of *α*- and *β*-himachalene by 5% of palladium on carbon (10%) gives, with good yield, the mixture of arylhimachalene and 1-isopropyl- 4,7-dimethylnaphthalene with respective proportions of 85/15. Treatment of the 1-isopropyl-4,7-dimethylnaphthalene by a mixture of nitric acid and sulfuric acid, gives the title compound with a yield of 70%. The structure of this new product was confirmed by its crystal structure (Fig. 1). Molecules are linked by a C9—H9···O2 contact (Table 1), forming a chain along [101] (Fig. 2).

### S2. Experimental

In a reactor of 250 ml equipped with a magnetic stirrer and a dropping funnel, we introduced 60 ml of dichloromethane, 3 ml of nitric acid and 5 ml of concentrated sulfuric acid. After cooling, added dropwise through the dropping funnel 6 g (30 mmol) of 1-isopropyl-4,7-dimethylnaphthalene dissolved in 30 ml of dichloromethane. The reaction mixture was stirred for 4 h, then added 50 ml of water ice and extracted with dichloromethane. The organic layers were combined, washed five times with 40 ml with water and dried over sodium sulfate and then concentrated under vacuum. The residue was subjected to chromatography on a column of silica gel with hexane-ethyl acetate (98/2) as eluent, to obtain 5 g (20 mmol) of the title compound which was recrystallized in hexane.

### S3. Refinement

All H atoms were fixed geometrically and treated as riding with C—H = 0.96 Å (methyl), 0.98 Å (methine) and 0.93 Å (aromatic), and with *U*_iso_(H) = 1.2*U*_eq_(aromatic and methine C) or 1.5*U*_eq_(methyl C).

### Crystal data

**Table:**

C_15_H_17_NO_2_	*F*(000) = 520
*M**_r_* = 243.30	*D*_x_ = 1.232 Mg m^−^^3^
Monoclinic, *P*2_1_/*n*	Mo *K*α radiation, λ = 0.71073 Å
*a* = 9.7637 (7) Å	Cell parameters from 2686 reflections
*b* = 12.6508 (9) Å	θ = 2.3–26.4°
*c* = 11.6162 (8) Å	µ = 0.08 mm^−^^1^
β = 113.897 (2)°	*T* = 296 K
*V* = 1311.82 (16) Å^3^	Box, colourless
*Z* = 4	0.45 × 0.35 × 0.30 mm

### Data collection

**Table:**

Bruker APEXII CCD diffractometer	2686 independent reflections
Radiation source: fine-focus sealed tube	2164 reflections with *I* > 2σ(*I*)
Graphite monochromator	*R*_int_ = 0.027
ω and φ scans	θ_max_ = 26.4°, θ_min_ = 2.3°
Absorption correction: multi-scan (*SADABS*; Bruker, 2009)	*h* = −11→12
*T*_min_ = 0.652, *T*_max_ = 0.746	*k* = −15→15
21437 measured reflections	*l* = −14→12

### Refinement

**Table:**

Refinement on *F*^2^	0 restraints
Least-squares matrix: full	Hydrogen site location: inferred from neighbouring sites
*R*[*F*^2^ > 2σ(*F*^2^)] = 0.047	H-atom parameters constrained
*wR*(*F*^2^) = 0.147	*w* = 1/[σ^2^(*F*_o_^2^) + (0.0738*P*)^2^ + 0.3258*P*] where *P* = (*F*_o_^2^ + 2*F*_c_^2^)/3
*S* = 1.07	(Δ/σ)_max_ = 0.001
2686 reflections	Δρ_max_ = 0.22 e Å^−^^3^
167 parameters	Δρ_min_ = −0.17 e Å^−^^3^

### Special details

**Table:**

Geometry. All e.s.d.'s (except the e.s.d. in the dihedral angle between two l.s. planes) are estimated using the full covariance matrix. The cell e.s.d.'s are taken into account individually in the estimation of e.s.d.'s in distances, angles and torsion angles; correlations between e.s.d.'s in cell parameters are only used when they are defined by crystal symmetry. An approximate (isotropic) treatment of cell e.s.d.'s is used for estimating e.s.d.'s involving l.s. planes.

### Fractional atomic coordinates and isotropic or equivalent isotropic displacement parameters (Å^2^)

**Table:**

	*x*	*y*	*z*	*U*_iso_*/*U*_eq_	
C1	0.48931 (16)	0.74540 (11)	0.55728 (13)	0.0410 (3)	
C2	0.53127 (17)	0.79068 (11)	0.46989 (14)	0.0458 (4)	
H2	0.4856	0.8530	0.4305	0.055*	
C3	0.64274 (16)	0.74433 (12)	0.43844 (13)	0.0434 (3)	
C4	0.71449 (15)	0.65180 (12)	0.48818 (13)	0.0426 (3)	
C5	0.66896 (14)	0.60094 (11)	0.57753 (12)	0.0391 (3)	
C6	0.73317 (18)	0.50374 (13)	0.63462 (15)	0.0506 (4)	
H6	0.8081	0.4735	0.6153	0.061*	
C7	0.68819 (19)	0.45344 (13)	0.71690 (15)	0.0540 (4)	
H7	0.7323	0.3894	0.7520	0.065*	
C8	0.57617 (17)	0.49648 (12)	0.74988 (13)	0.0464 (4)	
C9	0.51403 (16)	0.59105 (12)	0.69828 (13)	0.0428 (3)	
H9	0.4410	0.6204	0.7208	0.051*	
C10	0.55666 (14)	0.64644 (11)	0.61141 (12)	0.0370 (3)	
C11	0.37186 (18)	0.79765 (12)	0.59387 (16)	0.0509 (4)	
H11	0.3962	0.7793	0.6820	0.061*	
C12	0.2168 (2)	0.75490 (16)	0.5164 (2)	0.0741 (6)	
H12A	0.1910	0.7690	0.4289	0.111*	
H12B	0.1455	0.7886	0.5420	0.111*	
H12C	0.2155	0.6800	0.5293	0.111*	
C13	0.3728 (2)	0.91812 (14)	0.5855 (2)	0.0693 (5)	
H13A	0.4727	0.9438	0.6317	0.104*	
H13B	0.3074	0.9472	0.6207	0.104*	
H13C	0.3388	0.9391	0.4989	0.104*	
C14	0.82959 (18)	0.60027 (15)	0.45058 (17)	0.0592 (4)	
H14A	0.8302	0.6348	0.3772	0.089*	
H14B	0.8050	0.5270	0.4322	0.089*	
H14C	0.9268	0.6062	0.5184	0.089*	
C15	0.5277 (2)	0.43966 (15)	0.84114 (16)	0.0637 (5)	
H15A	0.5986	0.4530	0.9257	0.096*	
H15B	0.5228	0.3651	0.8247	0.096*	
H15C	0.4307	0.4647	0.8312	0.096*	
N1	0.67771 (18)	0.80420 (11)	0.34458 (13)	0.0576 (4)	
O1	0.5730 (2)	0.84079 (13)	0.25427 (14)	0.0865 (5)	
O2	0.80848 (18)	0.81719 (14)	0.36267 (14)	0.0869 (5)	

### Atomic displacement parameters (Å^2^)

**Table:**

	*U*^11^	*U*^22^	*U*^33^	*U*^12^	*U*^13^	*U*^23^
C1	0.0427 (7)	0.0414 (7)	0.0442 (7)	0.0013 (6)	0.0230 (6)	−0.0011 (6)
C2	0.0545 (8)	0.0401 (7)	0.0483 (8)	0.0027 (6)	0.0265 (7)	0.0034 (6)
C3	0.0503 (8)	0.0463 (8)	0.0409 (7)	−0.0084 (6)	0.0261 (6)	−0.0040 (6)
C4	0.0370 (7)	0.0527 (8)	0.0422 (7)	−0.0046 (6)	0.0204 (6)	−0.0091 (6)
C5	0.0344 (7)	0.0462 (8)	0.0368 (7)	0.0005 (5)	0.0145 (5)	−0.0035 (6)
C6	0.0465 (8)	0.0531 (9)	0.0529 (8)	0.0121 (6)	0.0209 (7)	0.0009 (7)
C7	0.0576 (9)	0.0491 (8)	0.0495 (8)	0.0092 (7)	0.0157 (7)	0.0083 (7)
C8	0.0489 (8)	0.0496 (8)	0.0381 (7)	−0.0055 (6)	0.0149 (6)	0.0019 (6)
C9	0.0432 (7)	0.0497 (8)	0.0403 (7)	−0.0003 (6)	0.0219 (6)	−0.0007 (6)
C10	0.0362 (6)	0.0408 (7)	0.0361 (7)	−0.0010 (5)	0.0167 (5)	−0.0027 (5)
C11	0.0559 (9)	0.0490 (9)	0.0586 (9)	0.0116 (7)	0.0344 (7)	0.0050 (7)
C12	0.0555 (10)	0.0673 (12)	0.1121 (16)	0.0006 (9)	0.0469 (11)	−0.0110 (11)
C13	0.0697 (11)	0.0513 (10)	0.0976 (14)	0.0112 (8)	0.0450 (11)	−0.0053 (9)
C14	0.0516 (9)	0.0752 (11)	0.0628 (10)	0.0041 (8)	0.0357 (8)	−0.0067 (8)
C15	0.0735 (11)	0.0662 (11)	0.0514 (9)	−0.0090 (9)	0.0253 (8)	0.0127 (8)
N1	0.0814 (10)	0.0541 (8)	0.0539 (8)	−0.0129 (7)	0.0444 (8)	−0.0072 (6)
O1	0.1158 (12)	0.0876 (10)	0.0662 (9)	0.0121 (9)	0.0474 (9)	0.0262 (8)
O2	0.0923 (10)	0.1066 (12)	0.0894 (10)	−0.0334 (9)	0.0653 (9)	−0.0057 (8)

### Geometric parameters (Å, º)

**Table:**

C1—C2	1.3651 (19)	C9—H9	0.9300
C1—C10	1.4353 (19)	C11—C12	1.514 (3)
C1—C11	1.5256 (19)	C11—C13	1.527 (2)
C2—C3	1.408 (2)	C11—H11	0.9800
C2—H2	0.9300	C12—H12A	0.9600
C3—C4	1.366 (2)	C12—H12B	0.9600
C3—N1	1.4767 (18)	C12—H12C	0.9600
C4—C5	1.4362 (19)	C13—H13A	0.9600
C4—C14	1.5087 (19)	C13—H13B	0.9600
C5—C6	1.417 (2)	C13—H13C	0.9600
C5—C10	1.4276 (18)	C14—H14A	0.9600
C6—C7	1.361 (2)	C14—H14B	0.9600
C6—H6	0.9300	C14—H14C	0.9600
C7—C8	1.407 (2)	C15—H15A	0.9600
C7—H7	0.9300	C15—H15B	0.9600
C8—C9	1.365 (2)	C15—H15C	0.9600
C8—C15	1.507 (2)	N1—O2	1.218 (2)
C9—C10	1.4223 (18)	N1—O1	1.221 (2)
			
C2—C1—C10	118.00 (12)	C1—C11—C13	112.97 (14)
C2—C1—C11	120.75 (13)	C12—C11—H11	107.3
C10—C1—C11	121.24 (12)	C1—C11—H11	107.3
C1—C2—C3	120.96 (13)	C13—C11—H11	107.3
C1—C2—H2	119.5	C11—C12—H12A	109.5
C3—C2—H2	119.5	C11—C12—H12B	109.5
C4—C3—C2	124.38 (13)	H12A—C12—H12B	109.5
C4—C3—N1	121.29 (13)	C11—C12—H12C	109.5
C2—C3—N1	114.33 (13)	H12A—C12—H12C	109.5
C3—C4—C5	115.69 (12)	H12B—C12—H12C	109.5
C3—C4—C14	124.21 (13)	C11—C13—H13A	109.5
C5—C4—C14	120.04 (14)	C11—C13—H13B	109.5
C6—C5—C10	117.68 (12)	H13A—C13—H13B	109.5
C6—C5—C4	121.27 (12)	C11—C13—H13C	109.5
C10—C5—C4	121.05 (13)	H13A—C13—H13C	109.5
C7—C6—C5	121.81 (14)	H13B—C13—H13C	109.5
C7—C6—H6	119.1	C4—C14—H14A	109.5
C5—C6—H6	119.1	C4—C14—H14B	109.5
C6—C7—C8	121.20 (14)	H14A—C14—H14B	109.5
C6—C7—H7	119.4	C4—C14—H14C	109.5
C8—C7—H7	119.4	H14A—C14—H14C	109.5
C9—C8—C7	118.42 (13)	H14B—C14—H14C	109.5
C9—C8—C15	121.09 (15)	C8—C15—H15A	109.5
C7—C8—C15	120.49 (15)	C8—C15—H15B	109.5
C8—C9—C10	122.51 (13)	H15A—C15—H15B	109.5
C8—C9—H9	118.7	C8—C15—H15C	109.5
C10—C9—H9	118.7	H15A—C15—H15C	109.5
C9—C10—C5	118.37 (12)	H15B—C15—H15C	109.5
C9—C10—C1	121.79 (12)	O2—N1—O1	123.46 (15)
C5—C10—C1	119.85 (12)	O2—N1—C3	118.79 (15)
C12—C11—C1	111.31 (13)	O1—N1—C3	117.71 (15)
C12—C11—C13	110.42 (15)		

### Hydrogen-bond geometry (Å, º)

**Table:**

*D*—H···*A*	*D*—H	H···*A*	*D*···*A*	*D*—H···*A*
C9—H9···O2^i^	0.93	2.60	3.4823 (18)	159

Symmetry code: (i) *x*−1/2, −*y*+3/2, *z*+1/2.
