# Supplementary figures and images for: Crystal structure of 1-isopropyl-4,7-dimethyl-3-nitro­naphthalene
Source: Acta Crystallogr E Crystallogr Commun. 2015 Aug 15;71(Pt 9):o659–60. doi: 10.1107/S2056989015014395 (PMC4555421; doi:10.1107/S2056989015014395)

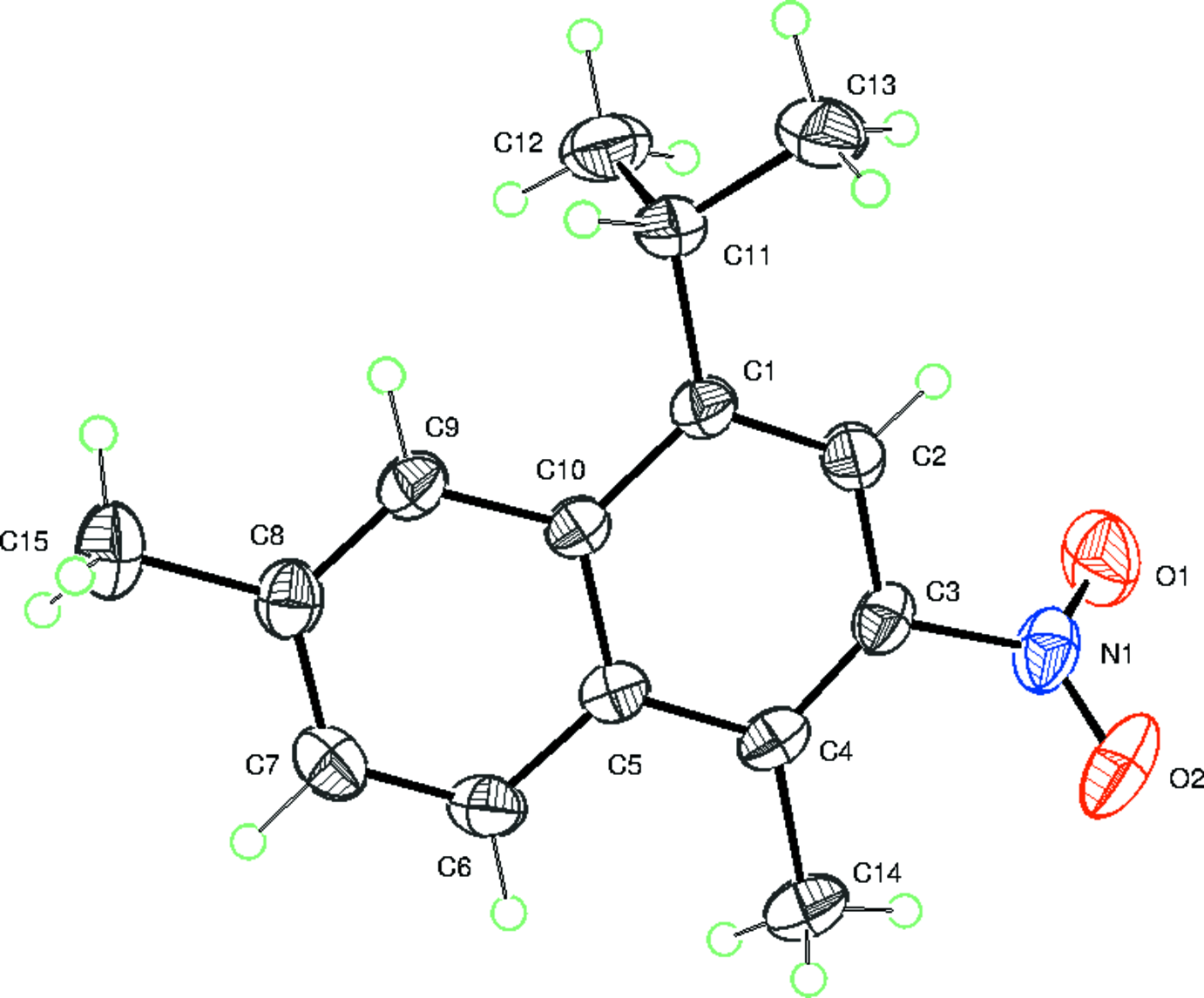

Supplement: Supplementary file 4 [file e-71-0o659-fig1.tif]

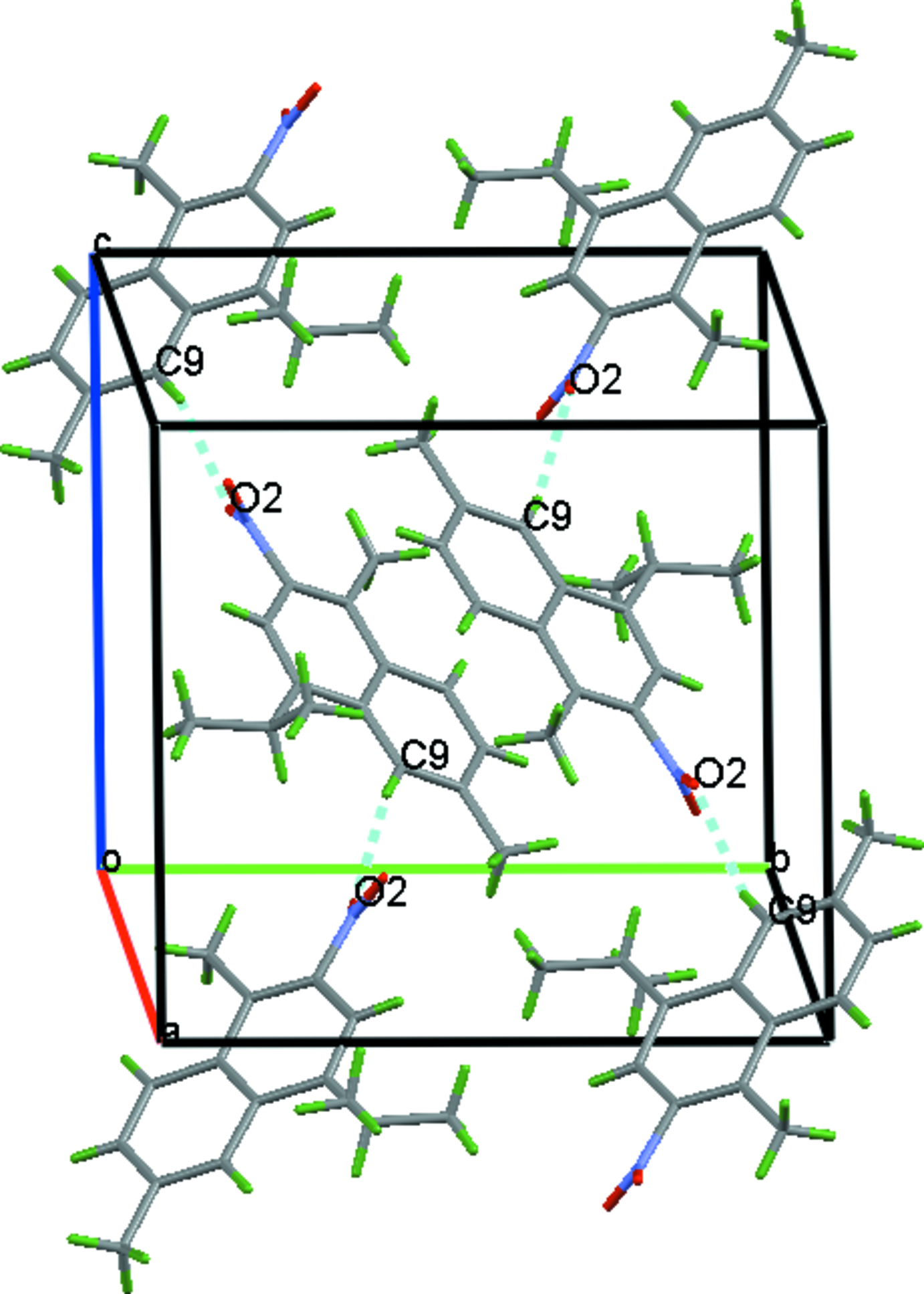

Supplement: Supplementary file 5 [file e-71-0o659-fig2.tif]
